# Invasive inflammatory fibroid polyp of the stomach: A case report and literature review

**DOI:** 10.1097/MD.0000000000041308

**Published:** 2025-02-14

**Authors:** Xingrong Yang, Sitong Guo, Ke Meng, Juan Tao

**Affiliations:** a Department of Pathology, The Second Hospital, Dalian Medical University, Dalian, Liaoning, China.

**Keywords:** gastritis cystica polyposa, inflammatory fibroid polyp, inverted hyperplastic polyp, platelet-derived growth factor receptor alpha mutation, stomach

## Abstract

**Rationale:**

Inflammatory fibrous polyp (IFP) is a distinct fibroblastic neoplasm with a predilection for the stomach and ileum. It usually presents prominent inflammatory infiltration, particularly eosinophils, and has been widely considered benign tumors without malignant biological behaviors. However, rare invasive cases have been reported.

**Patient concerns:**

A 75-year-old woman presented with unexplained hematemesis, dizziness, and weakness for 3 hours. Physical examination revealed upper abdominal tenderness on palpation.

**Diagnoses:**

Contrast-enhanced computed tomography revealed a 6 cm nodule with a high-density shadow and ring enhancement with well-defined borders in the gastric body. Gastroscopy showed multiple gastric polyps as well as a gastric submucosal mass with surface ulceration and mucosal disruption. Emergency laboratory results revealed anemia symptoms with an erythrocyte count of 2.63 × 10^12^/L and a hemoglobin level of 58.00 g/L. A laparoscopic distal gastrectomy was performed. The pathological results support the diagnosis of invasive IFP, and the tumor cells were infiltrated into gastric’s serosa layer.

**Interventions:**

The patient underwent laparoscopic distal gastrectomy resection. In addition, the patient received blood transfusion therapy for severe anemia, including Leukocyte privative red blood cell and Fresh frozen plasma.

**Outcomes:**

The patient was discharged home 2 weeks after surgery. There was no evidence of recurrence within the 4 years of surgery.

**Lessons:**

Except the common gastrointestinal stromal tumor and leiomyoma, IFP should also be considered by pathologists in the diagnosis of primary gastric non-epithelial tumor. Our case also emphasizes the invasive nature of IFP, a rare non benign biological feature (only 5 cases have been reported previously). Although it is very rare, it represents the potential development of the tumor, and should be paid attention to by pathologists and physicians. Otherwise, we report this case because of the first case of an IFP presenting with both gastritis cystica polyposa and inverted hyperplastic poly.

## 1. Introduction

Inflammatory fibrous polyp (IFP) was a rare type of mesenchymal tumor of the gastrointestinal tract and first reported by Vanek as “submucosal granuloma with eosinophilic infiltration” in 1949.^[1]^ It can affect individuals aged (1–84 years), and the median age lies in the fifth decade of life,^[2,3]^ and usually discovered found incidentally during endoscopy, whereas larger tumors may present with abdominal pain, bleeding, or obstruction. It predominantly occurs in the stomach (particularly the antrum) and ileum, and other sites, such as the anus,^[4]^ appendix,^[5]^ and gallbladder.^[6]^ An IFP is usually polypoid but can also be nonpolypoid.^[7]^ Patients’ symptoms are often related to the site and size of the tumor. Intussusception is a common presentation for small intestine tumors.

The pathogenesis of IFP can vary from reactive lesions with eosinophilic infiltration to neoplastic lesions with *platelet-derived growth factor receptor alpha (PDGFRA*) mutation. Even if it is a clonal rather than reactive lesion, it is classified as a benign tumor because it rarely has malignant biological behaviors like metastasis, infiltration, and recurrence. However, we report the second case of gastric primary invasive IFP, along with gastritis cystica polyposa (GCP) and inverted hyperplastic polyp (IHP) identified after a comprehensive differential diagnosis. Then we not only review its clinical features, diagnosis, tumorigenesis and cell type of origin but summarize the clinical and pathological features of other 4 invasive cases.^[3,8–11]^

## 2. Case presentation

### 2.1. Clinical features

A 75-year-old woman presented with unexplained hematemesis, dizziness, and weakness for 3 hours. She had a history of chronic gastritis and upper gastrointestinal hemorrhage, which led to the transfusion of deleukocyte suspension red blood cell 2 units, and pathologically confirmed hypertrophic polyps of the gastric fundus. No other specific past history (including the history of smoking or alcohol, heart disease or antiplatelet therapy) or family history was identified. Physical examination revealed upper abdominal tenderness on palpation. Her BMI value was 23.83.

### 2.2. Timeline and assistant examination

**Figure 1.**
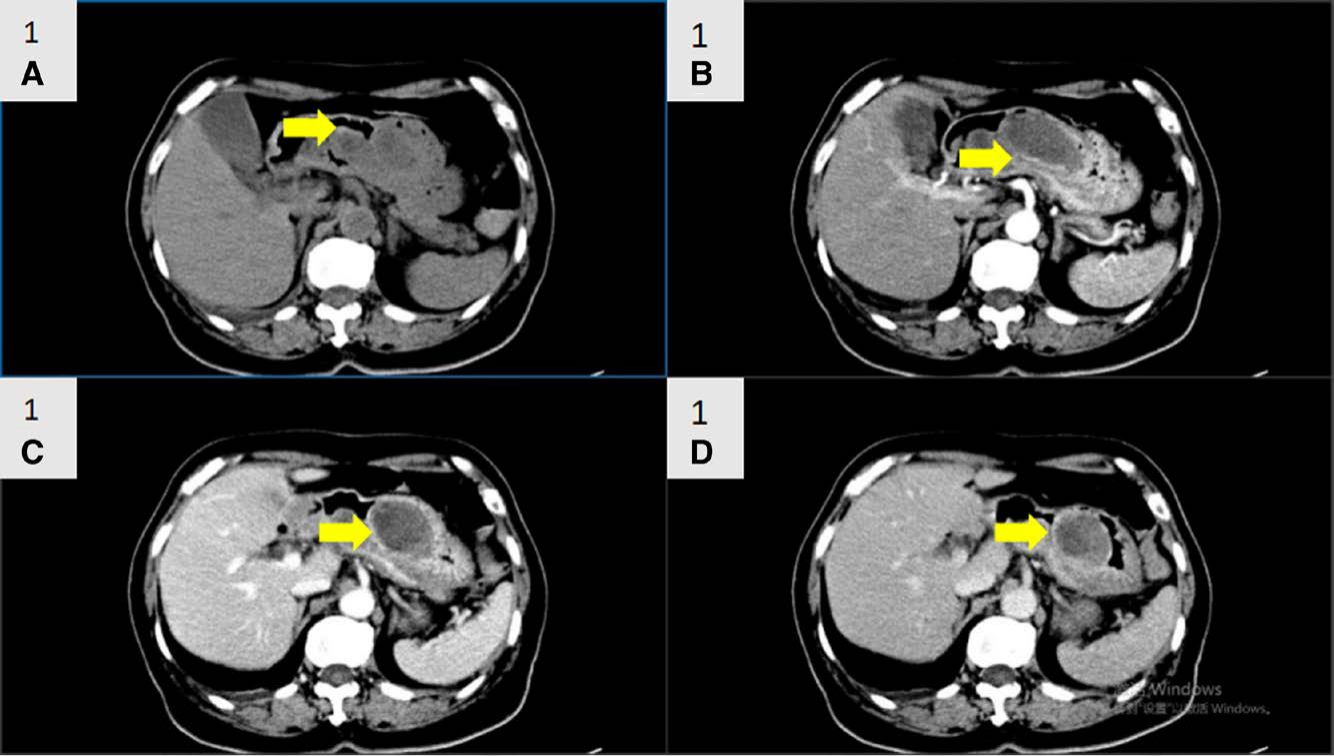
Contrast-enhanced computed tomography. (A–D) The CT revealed a nodule (yellow arrow) with high-density shadow in the gastric body, and ring enhancement in enhanced scan with clear boundaries.

**Figure 2.**
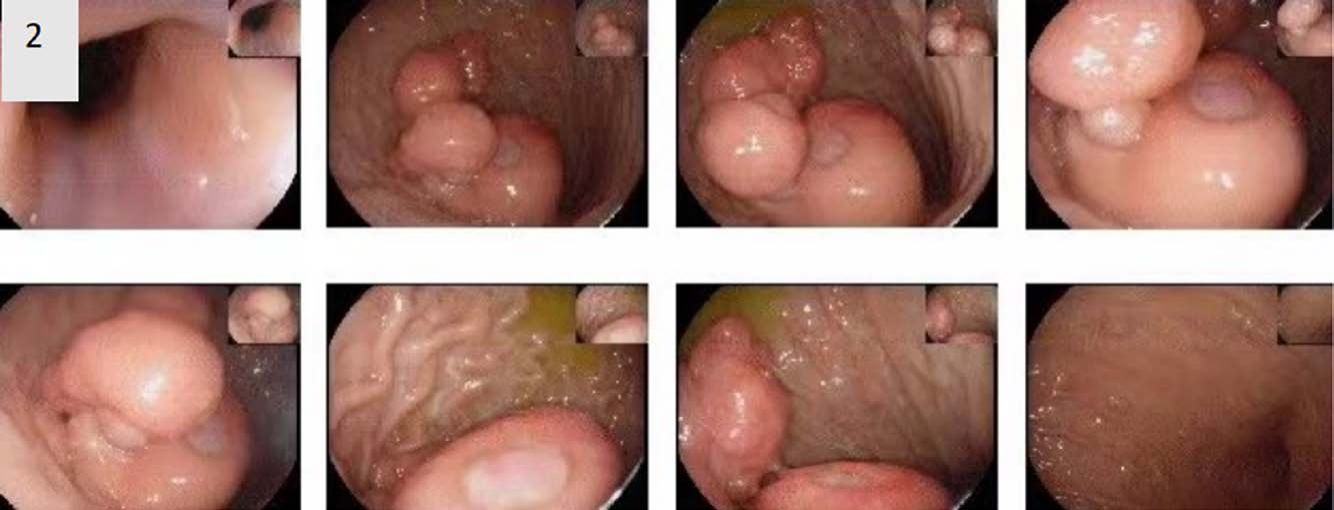
Gastroscopy. The gastroscopy showed multiple gastric polyps as well as a gastric submucosal mass with surface ulceration and mucosal disruption.

Emergency laboratory results revealed severe anemia symptoms with an erythrocyte count of 2.63 × 10^12^/L and a hemoglobin level of 58.00 g/L. Other blood biochemistry data were normal. Contrast-enhanced computed tomography revealed a nodule with a high-density shadow in the gastric body. The tumor had a maximum diameter of 6 cm, and the enhanced CT scan showed ring enhancement with well-defined borders (Fig. 1). Because of persistent upper gastrointestinal bleeding, the patient had severe anemia and could not have an immediate surgical treatment. Then, she received the medical hemostasis treatment for 5 days, including a blood transfusion treatment (leukocyte private red blood cell and fresh frozen plasma). Post transfusion examination demonstrated the erythrocyte count of 3.39 × 10^12^/L and a hemoglobin level of 83.00 g/L. After that, the gastroscopy showed multiple gastric polyps as well as a gastric submucosal mass with surface ulceration and mucosal disruption (Fig. 2). The endoscopist did not perform an endoscopic tumor biopsy due to avoid the recurrence of uncontrolled bleeding. Considering the large gastric mass and repeated history of bleeding, particularly with the older age of the patient, the possibility of malignancy and rebleeding was not ruled out, and a laparoscopic distal gastrectomy with Billroth II anastomosis was performed. The surgery lasted for 3 hours and the amount of bleeding was about 100 mL. The postoperative recovery was good, and no any perioperative complications occurred. The patient was discharged home 2 weeks after surgery. There was no evidence of recurrence within the 4 years of surgery.

### 2.3. Pathological results

Macroscopic appearance revealed a raised lobulated mass measuring 6 cm × 7 cm × 3.5 cm on the gastric mucosal surface, with a gray–white and gray–red surface and medium-to-tough texture. The tumor had evidently infiltrated into the muscularis propria layer. The partial cross-section of the mass was microcystic and contained clear fluid.

**Figure 3.**
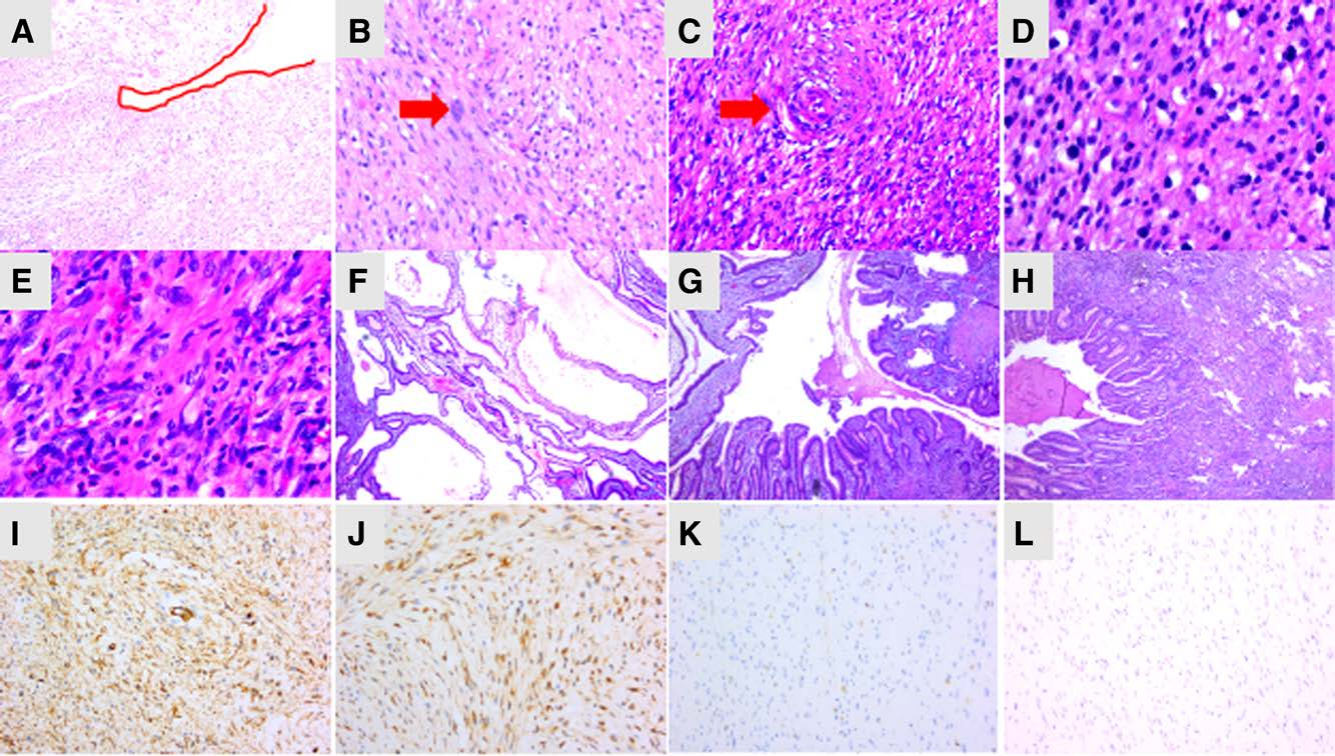
Hematoxylin-eosin staining. (A) Tumor cells infiltrated the serous layer of the gastric wall (100×). (B) Multiple nuclei cell (red arrow; 200×). (C) Spindle cells around the blood vessels showing a typical ‘onion-skin’ appearance (red arrow; 200×). (D) Greater infiltration of lymphocytes, plasma cells, and mast cells was noted in the stroma (400×). (E) Eosinophil infiltration was seen in the focal area (400×). (F) The dilated cystic wall is lined with a single columnar epithelium without cell atypia, and the cavity is filled with eosinophilic secretion and debris (40×). (G) Some of the glands were inverted into the submucosa, muscularis propria (40×). (H) Some of the glands were invaginated into the submucosa, muscularis propria (40×). (I) The tumor was diffusely strong expression of CD34 (200×). (J) The tumor was diffusely strong expression of PDGFRA (200×). (K) The tumor was negative of CD1a (200×). (L) The tumor was negative of Langerin (200×).

Hematoxylin–eosin staining revealed that the tumor realm was unclear and that the tumor center was mainly located in the mucosa and submucosa, infiltrating the muscularis propria and serosa of the gastric wall (Fig. 3A). The tumor mainly comprised moderate amounts of spindle and oval cells and relatively fewer heterotype or strange cells with large or multiple nuclei (Fig. 3B). Deep staining of these cells could be observed. Some cells showed non-pathological nuclear division, but necrosis was not observed. The tumor cells showed a diffuse, bundle, nodular, or vortex-like distribution with proliferation of blood vessels. In focal region, spindle cells around the blood vessels showed the typical ‘onion-skin’ appearance (Fig. 3C). Greater infiltration of lymphocytes, plasma cells, and mast cells was noted in the stroma (Fig. 3D). In particular, increased eosinophil infiltration was seen in the focal area (Fig. 3E), and focal myxoid stroma was observed. The surface of the mass was covered with gastric mucosa, and some of the glands were cystically dilated (Fig. 3E, F) and inverted into the submucosa, muscularis propria, and tumor stroma (Fig. 3G, H). The dilated cystic wall was lined with a single columnar epithelium without cell atypia, and the cavity was found to contain eosinophilic secretion and debris (Fig. 3E). The gastric pit epithelium without cell atypia showed evident hyperplasia and elongation with twisting, dilatation and distortion. Regional lymph node metastasis was not detected. A total of 22 lymph nodes were found in the adipose tissue around the major and minor bends, all of which were negative.

On immunohistochemistry, the tumor cells showed diffusely strong expression of CD34 (Fig. 3I) and PDGFRA (Fig. 3J); they were sporadically positive for S-100 protein and negative for CD117, DOG-1, smooth muscle actin, Caldesmon, ALK-80, ER, CD21, Bcl-2, STAT6, CD68, CD163, CD1a (Fig. 3K), and CD207 (langerin; Fig. 3L). The Ki-67 (MIB-1) labeling index in the spindle cells was <5%.

Genetic analysis revealed no mutation in the *KIT* gene (exons 9, 11, 12, 13, 14, 17, and 18), and *PDGFRA* exon 12, 14, and 18 mutations were not identified in our patient.

## 3. Discussion

Although the invasive features were uncommon and no exon mutations were detected in the PDGFRA gene of our patient, considering the patient’s clinical features and data as well as histopathology and immunohistochemistry findings, we still finally diagnosed her as having IFP. In this case, the tumor infiltrated the serosal layer, indicating some malignant potential. In the previous literature on IFP, only 6 cases (including our case) with “aggressive” features were reported. Therefore, we summarized the clinical and pathological features of the 6 invasive cases in the English literature available so far (Table 1, 2).^[3,8–11]^

**Table 1:** The summarize about clinical features and morphology of 6 invasive cases.

Case	Literature	Age	Sex	Site	Symptom	Size (cm)	Treatment	Follow up	Macroscopic findings	“Onion-skin” appearance	Myxoid stroma	Vascular network	Inflammatory cell infiltration	Depth
1	Bae JS’ literature (2015)	48	Female	Ileum	Abdominal pain	4.0	Surgery	12 mo	Myxoid texture/hemorrhagic	(−)	(+)	(+)	(+)	Subserosa
2	Shogo Tajima (2018)	70	Female	Ileum	Abdominal pain	4.0	Surgery	8 mo	Yellowish white/myxedematous texture	(−)	(+)	(+)	(+)	Serosa
3	Nova LM (2021)	46	Female	Ileum	Abdominal pain	3.5	Surgery	NK	Yellowish white/fibrous texture/ hemorrhagic	(+)	NK	(+)	(+)	Subserosa
4	Hirofumi Harima (2018)	62	Female	Stomach	Abdominal pain	2.7	Surgery	NK	NK	(−)	NK	(+)	(+)	Subserosa
5	Bangrong Xu (2024)	66	Female	Stomach	NK	2.0	Surgery	2 yr	NK	NK	NK	NK	NK	Subserosa
Our case	–	75	Female	Stomach	Hematemesis	7.0	Surgery	4 yr	Gray–white, gray–red/medium-to-tough texture	(+)	(+)	(+)	(+)	Serosa

(−) = negative, (+) = positive, NK = not known.

**Table 2:** The summarize about immunohistochemical and sequencing analysis and morphology of 6 invasive cases.

	Literature	Immunohistochemical										Sequencing analysis	
		CD34	PDGFRA	Vimentin	Keratin/AE1, AE3	SMA	S-100	CD117	DOG-1	ALK	Ki-67	c-kit	PDGFRA
Case 1	Bae JS’ report (2015)	(+)	(−)	(+)	(−)	(−)	(−)	(−)	(−)	NK	<10%	Exons (9, 11,13, 17) - wildtype	Exons (12,18)-wild-type
Case 2	Shogo ajima report (2018)	(+)	NK	(+)	NK	Focal (+)	(−)	NK	(−)	(−)	<1%	NK	Exon 12 mutation
Case 3	Nova LM report (2021)	(+)	NK	(+)	NK	(−)	(−)	NK	(−)	(−)	NK	NK	Exon 12 mutation
Case 4	Hirofumi Harima report (2018)	(+)	NK	NK	(−)	Focal (+)	(−)	(−)	NK	(−)	<1%	NK	Exons (12, 14, and 18)-wildtype
Case 5	Bangrong Xu (2024)	(+)	NK	(+)	NK	(−)	NK	(−)	(−)	NK	1%	NK	NK
Our case	–	(+)	(+)	NK	(−)	(−)	Sporadically (+)	(−)	(−)	(−)	<5%	Exons (9, 11, 12, 13, 14, 17, and 18) - wild-type	Exons (12 14, and 18)-wildtype

(−) = negative, (+) = positive, ALK = anaplastic lymphoma kinase, NK = not known, PDGFRA = platelet-derived growth factor receptor alpha, SMA = smooth muscle actin.

The clinical data of the patients are summarized in Table 1. There were 6 women aged 46 to 75 years (mean age, 61 years). Four patients presented with abdominal pain and 1 patient presented with hematemesis. We found that half of the tumors occurred in the stomach and the others in the ileum. The tumors ranged from 2.0 to 7.0 cm (mean size, 3.87 cm). All of them were treated with surgical resection. Among 4 cases with follow-up information, no recurrence or metastasis was found.

Grossly, 2 cases presented with a white and yellow surface (cases 2 and 3), and 2 cases have myxoid component, and 2 persons demonstrated this hemorrhagic surface. Histological findings are summarized in Table 1. The “onion-skin” appearance was only found in 2 of 5 patients. This showed that it was not a necessary feature for diagnosis, but it had a certain suggestive effect. Myxoid stroma was described in 3 patients. Five patients presented with vascular network and inflammatory cell infiltration (5/5). This suggested that they had an adequate nutrient supply and might be related to their aggressive nature. Notably, almost all of the tumors approached or even reached the serosa. However, no recurrence or metastasis was found in the 4 patients with follow-up information, which may be related to the limited follow-up time.

Immunohistochemical and genetic testing results are summarized in Table 2. Vimentin expression was noted in 4 tumors, and the remaining 2 tumors were negative for keratin or AE1/AE3. This indicated that they had characteristics of the stromal tumor differentiation. Among the 6 tumors, all expressed CD34 and were negative for CD117 or/and Dog-1. This results ruled out the possibility that they were gastrointestinal stromal tumors. Four cases tested negative for the anaplastic lymphoma kinase protein. The S100 protein was absent in 4 of 5 patients, and the remaining 1 patient showed only sporadic expression. This result initially ruled out the diagnosis of schwannoma. The Ki-67, labeling index, in the 4 cases was <10%, which indicated the malignancy of this tumor is not high in overall.

Among the 5 patients who detected *PAGFRA*, 2 harbored mutations in exon 12 of the *PDGFRA* gene, and mutations at any locus of the PDGFRA gene weren’t detected in the remaining cases. In 2 tumors containing information on kit gene testing, no mutations at any locus were detected. Notably, it was not until the discovery of familial cases that IFP began to be considered a possible tumor rather than a proliferative lesion.^[12]^ Actually, since Schildhaus et al observed that the tumor expressed PDGFRA in a series of 23 IFPs investigation in 2008, there were exons 12, 14, 15, and 18 have been reported in the later IFPs.^[13–15]^ However, as mentioned in the article, there were still many cases with negative PDGFRA gene mutation detection in the literature. We speculated the presence of other genetic mechanisms of PDGFRA for IFP, such as chromosomal rearrangements (FIP1L1-PDGFRA and ETV6-PDGFRB) in hematopoietic neoplasms, PDGFRA amplification and gene rearrangement between PDGFRA and VEGFR2 in pediatric forms of glioblastoma, and somatic mutation in PDGFRB in fusiform aneurysms or Penttinen premature aging syndrome.^[16]^

Researchers have long been committed to find the cell type of origin of IFP. Initially, IFPs were considered to be associated with GIST on the basis of morphology and positive CD34 expression. Eventually, different cell types were proposed as possible cells of origin of IFP, indicating possibilities of it having a neural, fibroblastic, myofibroblastic, histiocytic, fibrohistiocytic, or vascular origin.^[8]^ In 2004, in a study involving 16 cases, Pantanowitz et al^[17]^ proposed that IFPs have a dendritic cell origin with possible myofibroblastic differentiation. In 2018, based on the findings of a patient with IFPs and GISTs, Ricci et al reported that the stromal cells of IFPs express CD34 and PDGFRA, which are derived from telocytoma cells. They proposed the presence of a pathogenetic relationship between telocyte hyperplasia and both IFP and PDGFRA-mutant GIST. Furthermore, they reported using the term “telocytoma” for defining IFPs as it conveys both the pathogenetic (neoplastic) and histotypic (“telocytary”) essence of this tumor, unlike “IFP,” which rather indicates an inflammatory hyperplastic lesion.^[18]^ These previous studies have disproved the hypothesis that IFP is related to the origin of GIST, since GSIT originates from Cajal cells.^[19]^ However, further exploration is needed regarding the true cell of origin of IFP.

Besides, as this spindle tumor presented with typical morphological features like the “onion-skin” appearance and abundant eosinophils, it was not difficult to diagnose it. However, it was necessary to differentiate it from others. Gastrointestinal stromal tumor (GIST) should be the first alternative possibility when considering a differential diagnosis of an IFP because it also predominantly comprises spindle cells and shows high and diffuse expression for CD34, with a portion expressing PDGFRA as is also observed in IFP. However, in GIST, small-vessel proliferation is not so significant, and tumor cells do not show the “onion-skin” appearance around the blood vessels. Furthermore, eosinophil infiltration is typically not evident in the GIST background, and immunohistochemical markers not only positive for CD34 but CD117 and DOG-1. In addition, there are other diseases that need to be ruled out during differential diagnosis, such as inflammatory myofibroblastic tumor, solitary fibrous tumor, and histiocytes and dendritic cell-derived tumors.

Finally, what is unique to this case is that it combined deep cystic gastritis (GCP) and IHP. Firstly, GCP is a benign proliferative lesion, and its main pathogenesis is that chronic gastritis or ischemic changes leading to erosion of the gastric mucosa may interrupt the myometrium of the mucosa. Then, mucosal epithelial cells migrate toward the submucosa, and these cells and mucosal muscle layers begin to proliferate. Thus, the secretion from the submucosal glands cannot drain into the gastric cavity, and consequently, the glands passively expand.^[20,21]^ Based on this mechanism, we suspected that IFP growth under the mucosa triggered GCP formation in this case. In some reports, GCP has been considered as a high-risk factor for gastric cancer, and thus, its subsequent biological behavior and influence on patient prognosis need to be studied.^[22]^ Otherwise, IHP is an extremely rare gastric polyp characterized by the reverse growth of hyperplastic mucosal components, entry into the submucosa, and formation of hyperplastic polypoid lesions in the submucosa.^[23]^ Microscopically, the gastric pit shows evident hyperplasia and elongation as well as distorted and dilated deformation, and it contains hyperplasia components like pyloric glands and smooth muscle. The histological morphology of this case was consistent with the abovementioned characteristics, thus leading to a diagnosis of IHP.

## 4. Conclusion

Taken together, we herein reported a case of an IFP with increased infiltration presenting along with GCP and IHP. To our knowledge, this is the third case of an IFP invading the gastric mucosa, and it is the first case of an IFP co-occurring with GCP and IHP. Based on the rare invasive growth pattern of IFP, we reviewed 5 cases with invasive growth patterns in the literature. We speculate that the growth of IFP leads to the formation of GCP and IHP. Most IFPs are benign, but invasive growth is noted in rare cases, which might result in local recurrence with inadequate excision. Therefore, surgeons should have a comprehensive understanding of this rare entity to ensure optimal management and forecasting prognosis. Sure, it is also necessary that pathologists should report more cases to research the relationship between the invasive growth and poor biological behavior. In the future, more attention is needed to research the tumor nature of IFP, which should be studied in larger samples.

## Author contributions

**Formal analysis:** Sitong Guo.

**Investigation:** Sitong Guo, Ke Meng.

**Resources:** Ke Meng.

**Project administration:** Juan Tao.

**Supervision:** Juan Tao.

**Writing – original draft:** Xingrong Yang, Juan Tao.

**Writing – review & editing:** Xingrong Yang, Juan Tao.
